# Development and Validation of a Yoga-Based Intervention for Speech Dysfunction and Speech Difficulties Among Patients With Parkinson’s Disease

**DOI:** 10.7759/cureus.74287

**Published:** 2024-11-23

**Authors:** Parameshwar Some, Vijaykumar PS, Srinivas M, Nuzhath FJ

**Affiliations:** 1 Yoga and Life Sciences, S-VYASA (Swami Vivekananda Yoga Anusandhana Samsthana), Bangalore, IND; 2 Integrative Medicine, Kadamba Nano Care, Bangalore, IND; 3 Yoga Research, LYBL (Live Your Best Life), Los Angeles, USA; 4 Mind Management and Human Values, Human Networking Academy, JAIN (Deemed-to-be University), Bangalore, IND; 5 Integrative Medicine, Division of Yoga, Ministry of Ayush, Central Research Institute of Yoga and Naturopathy (CRIYN), Nagamangala, IND

**Keywords:** parkinson's disease, speech difficulties, validation, yoga module

## Abstract

Introduction

Parkinson's disease (PD) is primarily thought to be brought on by the death of dopaminergic neurons in the substantia nigra pars compacta of the basal ganglia. Communication difficulties are a common symptom of PD, affecting both motor speech and language systems. These challenges significantly impact the quality of life by reducing participation in communication, leading to social withdrawal, and increasing the risk of social isolation and stigma among individuals with PD. There are no established yoga protocols specifically addressing speech problems in PD. Hence, the research team aimed to develop and validate the yoga program targeting speech dysfunction and speech difficulties among patients with PD.

Materials and methods

The first part of this study involved developing a yoga-based intervention based on a review of classic literature and recently published research papers in the area of yoga and voice culture on PD. In the second step, 30 subject matter experts (SMEs) (yoga) confirmed the proposed module. The content-validity ratio (CVR) was determined using Lawshe's formula. The content validity was scored by these 30 SMEs in an independent manner on a three-point scale (0-2), with ‘not essential’ receiving 0, ‘useful but not essential’ receiving 1, and ‘essential’ receiving 2.

Results

Of the 68 practices selected for validation, 33 practices had a CVR score of ≥0.333, indicating high content validity. Thirty-five practices had a CVR score of <0.333, indicating low content validity. As a result, the final intervention module was created, consisting of 33 yoga practices with a CVR of 0.333.

Conclusion

A comprehensive and traditional literature-based yoga intervention module was developed and may be used as an intervention for speech difficulty in PD. Further studies are advocated to test the efficacy of the intervention.

## Introduction

Parkinson's disease (PD) is primarily thought to be brought on by the death of dopaminergic neurons in the substantia nigra pars compacta of the basal ganglia [1]. According to studies, up to 89% of people with PD develop speech symptoms [2]. Speech issues in PD have intricate neurological underpinnings. Communication difficulties are a common symptom of PD [3], affecting both motor speech and language systems. These challenges significantly impact the quality of life by reducing participation in communication, leading to social withdrawal, and increasing the risk of social isolation and stigma among individuals with PD. Vocal loudness reductions are partially ascribed to rigidity brought on by underlying dopaminergic insufficiency and hypokinesia, characterized by a decreased amplitude of movement [4].

Effective communication, which is essential for a high quality of life, requires intelligible speech and is a prerequisite for daily interactions. When a voice is thought to differ from those of the same age, gender, or ethnic group, it might become disordered due to differences in quality, pitch, loudness, flexibility, and vocal effort [4]. Of the more than 6,000,000 people with PD identified globally [5], speech dysarthria is another symptom of PD, characterized by ‘mono loudness,’ ‘monotone,’ and ‘unclear articulation’ [1]. People with PD may have difficulty speaking because they have difficulty coordinating the muscles responsible for breathing, articulation, resonance, and prosody [6]. For people with PD, functional communication is more important than motor speech impairment. And while functional communication is important for quality of life, it is not the only factor [7].

Acoustic parameters of speech production can be measured, and it has been proposed that these parameters can be measured objectively and noninvasively to assess symptomatic changes in PD [6]. Even after PD has been confirmed, a lack of detectable changes in speech and voice that the clinician can hear should not lead to the belief that there is no underlying issue. Acoustic and kinematic speech changes can be observed even in PD without dysarthria [8].

Despite the widespread occurrence of speech issues in PD, only a small percentage (3%-4%) seek speech treatment. Treatment options encompass pharmacological interventions, speech therapy, surgical procedures, deep brain stimulation, and vocal fold augmentation. Managing Parkinsonian dysarthria poses clinical challenges; however, integrating speech therapy into a multidisciplinary approach to patient care is crucial for addressing this aspect of the disease.

Research shows that exercise enhances both motor and non-motor control in patients with PD [9]. According to the American College of Sports Medicine (ACSM), yoga qualifies as exercise and offers additional benefits for participants [10]. Practicing yoga, whether at home or in a group setting, can help improve and maintain disability [11]. Integrating yoga with current medical treatments may optimize the benefits of managing disability in individuals with PD [12].

Yoga is a holistic mind-body practice that includes physical postures (asanas), breathing exercises (pranayama), and techniques for meditation and relaxation. It is widely recognized as a complementary and alternative medicine approach globally [13]. Practicing yoga has been shown to enhance gait, reduce fatigue, improve quality of life, and boost physical function in various neurological conditions [14].

Numerous studies have demonstrated the effectiveness of yoga in addressing both motor and non-motor symptoms associated with PD. In a randomized controlled trial involving 13 PD patients, the intervention group practiced Iyengar yoga for 60 minutes daily over 12 weeks. This group showed significant improvements in motor performance, balance, gait, and joint range of motion compared to the control group [15]. In a pre-post study with 10 adults with PD, participants attended a weekly 60-minute Hatha yoga session for eight weeks, resulting in notable reductions in anxiety and depression, as well as improvements in functional strength and leg flexibility [16]. In a similar study, 17 PD patients participated in a 10-week Iyengar yoga program, which included two-hour classes and daily 30-minute home practice sessions. Post-intervention, participants showed significant improvements in walking speed, physical performance, balance, and confidence in avoiding falls [17].

Research on the integration of yoga into speech-language therapy is limited [18]. A study was conducted on the use of modified yoga techniques in voice therapy with 55 participants, focusing on muscle tension dysphonia. The study found that yoga helped clients de-stress and concentrate before vocal exercises [19]. A single-subject case study was conducted incorporating asanas, pranayama, and meditation to treat a stroke patient with aphasia. Using the WAB-R (Western Aphasia Battery-Revised) measure, the patient showed a 9.1-point improvement in areas such as spontaneous speech, auditory verbal comprehension, repetition, and naming after yoga therapy, along with gains in reading and writing scores [20]. A study on unilateral nostril breathing (UNB) in post-stroke patients found that, after four weeks of guided instruction and six weeks of individual practice, participants showed improvements in affect and language. Self-reports indicated that UNB could be a cost-effective strategy to help alleviate certain post-stroke effects [21].

The above studies show that some have utilized tailored protocols designed and validated for their specific patient populations. However, there are no established yoga protocols specifically addressing speech problems in PD. Hence, the research team aimed to develop and validate the yoga program targeting speech dysfunction and speech difficulties among patients with PD.

## Materials and methods

The first part of this study involved developing a yoga-based intervention based on a review of classic literature and recently published research papers in the area of yoga and voice culture on PD. In the second step, 30 subject matter experts (SMEs) in yoga confirmed the proposed module. The content-validity ratio (CVR) was determined using Lawshe's formula [22].

Step 1

Under the guidance of well-known yoga masters, the researcher examined a variety of yoga scriptures to comprehend the basis for an integrated approach to yoga therapy and to create an evidence-based yoga intervention for voice culture in PD. Then, we collated the therapeutic corrective measures suggested by several texts (Patanjali Yoga Sutra, Hatha Yoga Pradipika, Hatharatnavali, Bhagavad Gita). The construction of a need-based table of practices for long-term holistic development in all five aspects of personality (the Pancha Kosha model of yoga) was based on the Hatha yoga literature. To create a list of all the methods used during this research, publications (books and research journal articles) on yoga for voice culture in PD and Parkinson’s rehabilitation were also researched. This yielded 68 practice items.

Step 2

To determine if a system, service, or product complies with standards and requirements for its intended purpose, Lawshe's CVR is used for validation [22]. Because there isn't a standardized yoga intervention for Parkinson’s rehabilitation, we came to the conclusion that there is a need for a tried-and-true, evidence-based yoga intervention for voice culture in PD. This led to the planning and execution of the present validation investigation.

Step 3

A focused group discussion (FGD) with 30 SMEs was organized to validate the 68-item intervention. The SMEs included Doctor of Medicine in yoga, Doctor of Philosophy in yoga with at least six to seven years of relevant experience, and yoga therapists (MSc in yoga) who have been continuously active in teaching the IAYT (Integrated Approach of Yoga Therapy) techniques to patients of all ages for more than eight years.

The content validity was scored by these 30 SMEs in an independent manner on a three-point scale (0-2), with ‘not essential’ receiving a score of 0, ‘useful but not essential’ receiving a score of 1, and ‘essential’ receiving a score of 2. Following validation, the data were analyzed using Lawshe's CVR [22].

Statistical analysis

Each of the 68 practices was evaluated by 30 SMEs. For all 68 items, Lawshe's CVR was computed using the formula [22]:

\[ \text{CVR} = \frac{N_e - \frac{N}{2}}{\frac{N}{2}} \]

In the formula, ‘N_e_’ is the number of SME panelists who indicated ‘essential,’ and ‘N’ is the total number of SME panelists. According to Lawshe's significance table, the value of CVR for 33 SMEs is equal to 0.333, meaning that all items with CVR > 0.333 are appropriate and necessary for the module.

## Results

CVR was established for each of the 68 yoga practices (Table 1). At an FGD, all practices that met the cut-off CVR for 30 experts were addressed and accepted by all participants.

**Table 1:** CVR for each practice on the initial Yogic Breathing Intervention (YBI) list. CVR, Content validity ratio

Practice List	CVR
Hand Stretch Breathing	-0.266
Tiger Breathing	0.733
Dog Breathing	0.6
Tiger Stretch	0.06
Tongue In and Out	0.866
Tongue Up and Down	0.933
Tongue Right and Left	0.933
Tongue Rotation	0.9
Tongue Massage	0.8
Lip Front and Back	0.8
Lip Side-to-Side Movement	0.6
Jaw Up and Down Movement	0.6
Jaw Side-by-Side Movement	0.733
Jogging	-0.8
Forward and Backward Bending	-0.6
Side Bending	-0.33
Pawanmuktasana Kriya	-0.4
Head Rolling	0.06
Trunk Twisting	-0.33
Butterfly	-0.53
Straight Leg Raising	-0.53
Alternate Toe Touching	-0.53
Drill Walking	-0.266
Padanguli Naman (Toe Stretching)	-0.266
Mani Bandha Naman (Wrist Bending)	-0.266
Kehuni Naman (Elbow Stretching)	-0.33
Greevasanchalana (Neck Movements)	0.4
Scandasanchalan (Shoulder Movements)	0
Katisanchalana (Waist Movements)	-0.4
Janusanchalana (Knee Movements)	-0.53
Padasanchalana (Ankle Movements)	-0.53
Smarana Shakti Vikasaka	0.266
Anguli Shakti Vikasaka (Fingers)	-0.46
Angula Mula Shakti Vikasaka (Root of Fingers)	-0.6
Mani-Bandha Shakti Vikasaka (Wrists)	-0.46
Netra Sakti Vikasaka (Eye)	-0.066
Griva Shakti Vikasaka (Neck)	0.4
Karna Shakti Vikasaka (Ear)	0.2
Kara Prastha Shakti Vikasaka (Back of Hand)	-0.33
Jangha Shakti Vikasaka (Thighs)	-0.6
Kati Shakti Vikasaka (Forward, Backward and Side Bending)	-0.53
Pada Mula Shakti Vikasaka (Ankle)	-0.53
Gulpha, Pada Prista, Pada Tala Shakti Vikasaka (Feet)	-0.46
Ardha Kati Chakrasana	0.2
Ardha Chakrasana	0.4
Pada Hastasana	0.266
Ushtrasana	0.666
Bhujangasana	0.533
Dhanurasana	0.2
Viparita Karani	0.4
Matsyasana	0.333
Halasana	0.4
Sectional Breathing	0.8
Bhramari (With Shanmukhi Mudra and Jhalandhar Bandh)	0.866
Bhramara (With Shanmukhi Mudra and Jhalandhar Bandh)	0.8
Ujjayi	0.933
Nadishuddhi	0.8
Simha Mudra	0.866
Shloka Chanting	1
Lax Vox	0.53
Loud Chanting of A, U, M, AUM, OM	1
Nadanusandana	0.933
Om Meditation	0.533
Cyclic Meditation	0.4
Jala Neti	0.4
Sutra Neti	0.2
Vamana Dhauti	0.53
Trataka	-0.13

Of the 68 practices selected for validation, 33 practices had a CVR score of ≥0.333, indicating high content validity (Table 2). Thirty-five practices (Table 3) had a CVR score of <0.333, indicating low content validity. As a result, the final intervention module was created, consisting of 33 yoga practices with a CVR of 0.333.

**Table 2:** Yoga intervention practices with CVR ≥ 0.333. CVR, Content validity ratio

Practice List	CVR
Tiger Breathing	0.733
Dog Breathing	0.6
Tongue In and Out	0.866
Tongue Up and Down	0.933
Tongue Right and Left	0.933
Tongue Rotation	0.9
Tongue Massage	0.8
Lip Front and Back	0.8
Lip Side-to-Side Movement	0.6
Jaw Up and Down Movement	0.6
Jaw Side-by-Side Movement	0.733
Greevasanchalana (Neck Movements)	0.4
Griva Shakti Vikasaka (Neck)	0.4
Ardha Chakrasana	0.4
Ushtrasana	0.666
Bhujangasana	0.533
Viparita Karani	0.4
Matsyasana	0.333
Halasana	0.4
Sectional Breathing	0.8
Bhramari (With Shanmukhi Mudra and Jhalandhar Bandh)	0.866
Bhramara (With Shanmukhi Mudra and Jhalandhar Bandh)	0.8
Ujjayi	0.933
Nadishuddhi	0.8
Simha Mudra	0.866
Shloka Chanting	1
Lax Vox	0.53
Loud Chanting of A, U, M, AUM, OM	1
Nadanusandana	0.933
Om Meditation	0.533
Cyclic Meditation	0.4
Jala Neti	0.4
Vamana Dhauti	0.53

**Table 3:** Yoga intervention practices with CVR ≤ 0.333. CVR, Content validity ratio

Practice List	CVR
Hand Stretch Breathing	-0.266
Tiger Stretch	0.06
Jogging	-0.8
Forward and Backward Bending	-0.6
Side Bending	-0.33
Pawanmuktasana Kriya	-0.4
Head Rolling	0.06
Trunk Twisting	-0.33
Butterfly	-0.53
Straight Leg Raising	-0.53
Alternate Toe Touching	-0.53
Drill Walking	-0.266
Padanguli Naman (Toe Stretching)	-0.266
Mani Bandha Naman (Wrist Bending)	-0.266
Kehuni Naman (Elbow Stretching)	-0.33
Scandasanchalan (Shoulder Movements)	0
Katisanchalana(Waist Movements)	-0.4
Janusanchalana (Knee Movements)	-0.53
Padasanchalana (Ankle Movements)	-0.53
Smarana Shakti Vikasaka	0.266
Anguli Shakti Vikasaka (Fingers)	-0.46
Angula Mula Shakti Vikasaka (Root of Fingers)	-0.6
Mani-Bandha Shakti Vikasaka (Wrists)	-0.46
Netra Sakti Vikasaka (Eye)	-0.066
Karna Shakti Vikasaka (Ear)	0.2
Kara Prastha Shakti Vikasaka (Back of Hand)	-0.33
Jangha Shakti Vikasaka (Thighs)	-0.6
Kati Shakti Vikasaka (Forward, Backward, and Side Bending)	-0.53
Pada Mula Shakti Vikasaka (Ankle)	-0.53
Gulpha, Pada Prista, Pada Tala Shakti Vikasaka (Feet)	-0.46
Ardha Kati Chakrasana	0.2
Pada Hastasana	0.266
Dhanurasana	0.2
Sutra Neti	0.2
Trataka	-0.13

## Discussion

The current study focused on the development and validation of a yoga module for speech dysfunction and speech difficulties among patients with PD. A 33-item yoga module was specifically created to address these speech difficulties. The beneficial effects of yoga for PD have been well documented in multiple clinical trials. The experts found that the module, as a whole, was acceptable and capable of achieving its intended goal. Most of them recommended one hour of yoga practice at least three days a week. To improve adherence, they also suggested one supervised session every month following an initial training period of 10 sessions over six months.

There are few studies available on the development and validation of yoga modules for common conditions. Common approaches in these studies typically include: (1) reviewing yoga texts (literary review), (2) validation by yoga experts, and (3) administering the module in a small sample size. For example, a study focused on a yoga module for PD conducted validation with 20 experts, where 21 out of 28 techniques achieved the required CVR ratio [13]. Another study, developing a yoga module for depression, reported validation by nine experts and conducted a pilot intervention study with seven patients [23]. In a study developing an IAYT yoga module for obesity in adolescents, the module was developed based on a literary review and validated by 16 yoga experts, comprising 43 techniques [24]. Similarly, a study creating a yoga module for children with visual impairment developed the module from traditional texts, validated it with 25 yoga experts, and piloted it with nine children [25].

The current study follows a similar approach in developing the yoga module; however, it is considered a pioneering step in creating a holistic and integrative module specifically designed to address speech difficulties in PD participants. To the best of our knowledge, this study represents the first concerted effort toward developing such a focused module for individuals with PD. The strength of the module lies in its specific techniques designed to achieve this goal. Utmost care was taken in its design to ensure it is suitable for all PD participants, considering their limited flexibility and mobility issues.

The novelty of this study lies in its development of a framework that provides justification and seeks validation from experts. This framework for speech difficulties in PD participants, validated by experienced yoga experts, is the first of its kind. The expert panel included an experienced Doctor of Medicine in yoga, Doctor of Philosophy in yoga with at least six to seven years of relevant experience, and yoga therapists (MSc in yoga) who have been actively teaching IAYT techniques to patients of all ages for more than eight years. Compared with related works, the yoga modules include loosening exercises, specific asanas, breathing practices, and relaxation techniques tailored to PD patients. Validation involved assessing content validity using expert panels, with practices like alternate nostril breathing (Nadi Shodhana) and specific postures showing high validity scores. Such modules aim to address motor and non-motor symptoms of PD. Out of the 68 yogic techniques suggested, 33 received high CVR scores in expert validation, confirming the high content validity of the module.

The final practices in the developed module include breathing exercises and pranayama, which aid in relaxation, improve blood flow, and assist in managing motor difficulties in PD [26]. The loosening practices (Shithilikarna Vyayamas) and facial exercises included in this module help loosen the facial joints and muscles, reducing stiffness and promoting easier mobility. All the asanas chosen in the final list are known to enhance blood circulation to the face and neck region and aid in improving nerve conduction [15]. The mudras and kriyas used here help soothe the facial nerves and relax the muscles. Nadanusandhana and chanting aid in voice culture, as recommended in traditional texts. Meditation and relaxation help reduce stress and anxiety, thereby improving the relaxation of the body and mind, which facilitates the alleviation of anxiety, depression, and stress related to PD [16]. 

The limitations include the adaptability, feasibility, and validity of yoga practices to suit the varying abilities of individuals with PD, due to their progressive mobility. Future studies must determine whether the developed yoga module is feasible and effective for individuals with PD.

Clinical practice and literature suggest that a yoga-based lifestyle has the potential to improve speech difficulties in various conditions, such as stroke. Similarly, yoga may offer a comparable effect in addressing speech difficulties in individuals with PD. Future studies should explore the efficacy of this yoga module through feasibility testing among patients with PD who experience speech difficulties, alongside conventional care. The module should be evaluated using objective variables, such as sound pressure level, in a long-term study. Efforts should focus on determining the optimal duration, frequency of practices, and logical sequencing of exercises during the feasibility testing phase. It is advisable to conduct in-person teaching of the yoga module to foster a sense of community and mitigate the risk of injuries in PD participants.

## Conclusions

A comprehensive and traditional literature-based yoga intervention module was developed to facilitate recovery from the multidimensional symptoms of PD with voice difficulty. The yoga module was validated by 30 experts. The final module could be used as an intervention for speech difficulties in PD; however, further studies are advocated to test the efficacy of the intervention.

## References

[1] Kwan LC, Whitehill TL (2011). Perception of speech by individuals with Parkinson's disease: a review. Parkinsons Dis.

[2] Ramig L, Halpern A, Spielman J, Fox C, Freeman K (2018). Speech treatment in Parkinson's disease: randomized controlled trial (RCT). Mov Disord.

[3] Barnish MS, Barran SM (2020). A systematic review of active group-based dance, singing, music therapy and theatrical interventions for quality of life, functional communication, speech, motor function and cognitive status in people with Parkinson's disease. BMC Neurol.

[4] Payten CL, Chiapello G, Weir KA, Madill CJ (2024). Frameworks, terminology and definitions used for the classification of voice disorders: a scoping review. J Voice.

[5] Levy ES, Moya-Galé G, Chang YH, Freeman K, Forrest K, Brin MF, Ramig LA (2020). The effects of intensive speech treatment on intelligibility in Parkinson's disease: a randomised controlled trial. EClinicalMedicine.

[6] Dimauro G, Nicola VDI, Bevilacqua V, Caivano D, Girardi F (2017). Assessment of speech intelligibility in Parkinson's disease using a speech-to-text system. IEEE Access.

[7] Barnish MS, Horton SM, Butterfint ZR, Clark AB, Atkinson RA, Deane KH (2017). Speech and communication in Parkinson's disease: a cross-sectional exploratory study in the UK. BMJ Open.

[8] Miller N (2012). Speech, voice and language in Parkinson's disease: changes and interventions. Neurodegenerative Disease Management.

[9] van der Kolk NM, King LA (2013). Effects of exercise on mobility in people with Parkinson's disease. Mov Disord.

[10] Ross A, Thomas S (2010). The health benefits of yoga and exercise: a review of comparison studies. J Altern Complement Med.

[11] Ni M, Signorile JF, Mooney K (2016). Comparative effect of power training and high-speed yoga on motor function in older patients with Parkinson disease. Arch Phys Med Rehabil.

[12] Mooventhan A, Nivethitha L (2017). Evidence based effects of yoga in neurological disorders. J Clin Neurosci.

[13] Kakde N, Metri KG, Varambally S, Nagaratna R, Nagendra HR (2017). Development and validation of a yoga module for Parkinson disease. J Complement Integr Med.

[14] Mishra SK, Singh P, Bunch SJ, Zhang R (2012). The therapeutic value of yoga in neurological disorders. Ann Indian Acad Neurol.

[15] Colgrove YS, Sharma N, Kluding P (2012). Effect of yoga on motor function in people with Parkinson’s disease: a randomized, controlled pilot study. J Yoga Phys Ther.

[16] Roland KP (2014). Applications of yoga in Parkinson’s disease: a systematic literature review. J Parkinsonism Restless Legs Synd.

[17] Lee LW (2006). The effect of yoga exercises on balance, lower-extremity function and gait in people with Parkinson’s disease. Arch Physical Med Rehabil.

[18] Longtin ES, Fitzpatrick JA, Mozes M (2017). Yoga for Speech-Language Development. https://www.barnesandnoble.com/w/yoga-for-speech-language-development-susan-e-longtin/1124808079.

[19] Moore C (2012). Reflections on clinical applications of yoga in voice therapy with MTD. Logoped Phoniatr Vocol.

[20] Rivkin N (2013). The Effects of Yoga on Aphasia Rehabilitation. https://www.semanticscholar.org/paper/The-Effects-of-Yoga-on-Aphasia-Rehabilitation-Rivkin/f7cc3f273f1f21ab5c6a892943388c27a682b70e.

[21] Marshall RS, Basilakos A, Williams T, Love-Myers K (2014). Exploring the benefits of unilateral nostril breathing practice post-stroke: attention, language, spatial abilities, depression, and anxiety. J Altern Complement Med.

[22] Lawshe CH (1975). A quantitative approach to content validity. Pers Psychol.

[23] Naveen GH, Rao MG, Vishal V, Thirthalli J, Varambally S, Gangadhar BN (2013). Development and feasibility of yoga therapy module for out-patients with depression in India. Indian J Psychiatry.

[24] Rathi SS, Raghuaram N, Tekur P, Joshi RR, Ramarao NH (2018). Development and validation of integrated yoga module for obesity in adolescents. Int J Yoga.

[25] Mohanty S, Venkataramanujam S, Pradhan B, Hankey A (2019). Development and validation of a yoga module for children with visual impairment: a feasibility study. Br J Vis Impair.

[26] Jain R, Parmar R, Jain NK (2022). Role of yogic management in Parkinson’s disease (Kampvata). TUJ Homo & Medi Sci.

